# An arthropod transporter IsOATP4056 exerts inhibitory effect on its interacting membrane protein to facilitate rickettsial pathogen survival in ticks

**DOI:** 10.1186/s12964-025-02545-w

**Published:** 2025-11-22

**Authors:** P. P. Mahesh, Hameeda Sultana, Girish Neelakanta

**Affiliations:** Department of Biomedical and Diagnostic Sciences, College of Veterinary Medicine, University of Tennessee, Knoxville, TN 37996 USA

**Keywords:** Human anaplasmosis, Anaplasma phagocytophilum, Ixodes scapularis, Organic anion transporting polypeptide (OATP), AhR, Hypothetical protein, Xanthurenic acid (XA), Protein-protein interactions, Arthropod innate immunity, Bacterial survival

## Abstract

**Background:**

The black-legged tick, *Ixodes scapularis*, transmits several medically important pathogens to humans including *Anaplasma phagocytophilum*. Our previous studies provided evidence that this bacterium modulates arthropod organic anion transporting polypeptide (IsOATP4056) and tryptophan pathways for its survival and transmission from ticks. In this study, we identified that IsOATP4056 interacts with *I. scapularis* hypothetical protein (IsHP) to suppress arthropod innate immunity thereby facilitating *A. phagocytophilum* survival in ticks and tick cells.

**Methods:**

Co-precipitation with recombinant IsHP and tick protein lysates followed by immunoblotting analysis with anti- IsOATP4056 antibody was performed to reveal whether these two proteins directly interact. RNAi-mediated silencing experiments were performed to understand the roles of IsHP, IsOATP4056 and Aryl hydrocarbon Receptor (AhR) in tick-*A. phagocytophilum* interactions.

**Results:**

Immunoprecipitation and immunoblotting analysis revealed interaction of IsHP with IsOATP4056. RNAi-mediated silencing of *ishp* expression affected arthropod innate immune response that resulted in significantly increased bacterial burden in tick cells. In contrast, RNAi-mediated silencing of *isoatp4056* expression or antibody-mediated blocking of IsOATP4056 upregulated *ishp* and innate immune response that controlled bacterial burden in ticks and tick cells. Furthermore, we noted that *A. phagocytophilum* infection or treatment with tryptophan metabolite xanthurenic acid (XA) significantly upregulates Aryl hydrocarbon Receptor (AhR) expression in ticks and tick cells. RNAi-mediated silencing of *ahr* expression decreased *isoatp4056* transcripts and bacterial burden but increased the *ishp* expression. EMSA results further support that AhR and XA positively regulate *isoatp4056* promoter.

**Conclusions:**

These results elucidate that *A. phagocytophilum* infection triggers AhR-mediated regulation of *isoatp4056* expression to inactivate IsHP-associated *pelle* expression for its survival in ticks and tick cells.

**Supplementary Information:**

The online version contains supplementary material available at 10.1186/s12964-025-02545-w.

## Introduction

Ticks are important vectors for pathogens that causes human diseases like Lyme borreliosis, human anaplasmosis, ehrlichiosis, rickettsioses, tularemia, Powassan encephalitis, tick-borne encephalitis, Kyasanur forest disease and several others [1–7]. Human anaplasmosis is caused by an obligate intracellular bacterium, *Anaplasma phagocytophilum* [4, 8, 9]. The symptoms of the disease include fever, malaise, headache, arthralgia, thrombocytopenia, and leukopenia [4, 8]. *Anaplasma phagocytophilum* is transmitted by a black legged tick, *Ixodes scapularis* [4, 8, 10–12]. The developmental stages of these ticks include eggs, larvae, nymphs, and adults [12–14]. Larvae or nymphs acquire *A. phagocytophilum* from an infected vertebrate host. Upon entering larvae or nymphs, this bacterium can undergo transstadial transmission to nymphs and adult stages of ticks, respectively [12–14]. However, this bacterium cannot be transovarially transmitted from adults to eggs [12–14]. In addition to humans, *A. phagocytophilum* also infects several animals such as horses, dogs, deer, and cattle [11, 15, 16]. As soon as *A. phagocytophilum* enters a host cells, the bacterium forms a host derived vacuole, generally referred as morula, where it resides and multiplies [10, 11]. This vacuole was noted to be intact throughout the infection cycle of this bacterium in the host cells [10, 11]. Several studies have reported that *Anaplasma phagocytophilum* subverts host cell signaling both in arthropods and vertebrates for its survival in these different organisms [10, 11, 17–31].

Organic Anion Transporting Polypeptides (OATPs) are transmembrane proteins that are involved in transport of various molecules including hormones, signaling molecules and drugs [32–36]. *Ixodes scapularis* ticks encode nine OATPs that are expressed in various tick tissues [31, 37]. In a previous study, we reported that one of the *I. scapularis* OATPs (IsOATP4056) was upregulated both at transcript and protein levels upon *A. phagocytophilum* infection of ticks and tick cells [25, 31]. In addition, we noted that exogeneous addition of a tryptophan pathway metabolite, xanthurenic acid (XA), to ticks and tick cells resulted in an increase of both the expression of *isoatp4056* and the bacterial burden [31]. Among the nine OATPs in ticks, only *isoatp4056* was upregulated in salivary glands during *A. phagocytophilum* infection [31]. RNAi-mediated knockdown of *isoatp4056* and treatment with OATP inhibitor reduced the bacterial replication in ticks and tick cells [31]. In another study, we noted that inhibition of OATPs affected the viral burden of tick-borne Langat virus (LGTV) in tick cells [28]. We also reported that XA can help the pathogen to evade reactive oxygen species [24, 26]. Another study from our laboratory showed that *A. phagocytophilum* downregulates microRNA133 (miR-133) that targets *isoatp4056* mRNA [27]. In the same study, we also noted that exogenous microinjection of miR-133 in to ticks affected transmission of *A. phagocytophilum* from arthropod to the vertebrate host [27]. Furthermore, we reported that *A. phagocytophilum* increases the endogenous levels of XA in ticks and tick cells, and this facilitated increase in the viability of tick cells [26]. Collectively, these studies highlight that IsOATP4056 is critical for *A. phagocytophilum* survival and transmission from infected ticks to the naïve vertebrate host.

In a recent study, we showed that passive immunization of mice with an antibody targeting C-terminal extracellular loop (anti-EL6) of IsOATP4056 impaired transmission of *A. phagocytophilum* from ticks to murine host and affected the molting process in ticks [25]. In the same study, we also noted an increased cell death in tick cells upon treatment with EL-6 antibody [25]. In another recent study, we reported that the ortholog of IsOATP4056 in *Haemaphysalis longicornis* ticks was upregulated upon *A. phagocytophilum* infection and treatment of these ticks with EL-6 antibody affected the bacterial burden [38]. We noted that blocking of IsOATP4056 with EL-6 antibody resulted in the activation of some of the *I. scapularis* innate immune genes that affected the bacterial survival in ticks [25]. The mechanism by which IsOATP4056 is involved in suppressing tick innate immune pathways was not understood. In this study, we provide evidence that IsOATP4056 interacts with tick membrane hypothetical protein, designated as IsHP, to suppress the arthropod innate immunity that facilitates *A. phagocytophilum* survival in ticks. In addition, we identified that XA and arthropod Aryl hydrocarbon Receptor (AhR) are involved in the positive regulation of *isoatp4056* expression and negative regulation of *ishp* expression. These studies provide detailed mechanistic insights on how arthropod IsOATP4056, and tryptophan pathway facilitates rickettsial pathogen survival in ticks.

## Methods

### Bacterial isolates, ticks, and tick cell line

*Anaplasma phagocytophilum* strain NCH-1 was used throughout this study. This strain was obtained from BEI Resources, NIAID, NIH. *Anaplasma phagocytophilum* was maintained in the human promyelocytic cell line (HL-60 cells) as described in our previous studies [25, 26, 31]. The HL-60 cell line was obtained from ATCC, USA. C57BL/6J mice (Jackson laboratory, USA) were used in this study. *Anaplasma phagocytophilum* infection was maintained in B6.129S7-Rag1tm1Mom/J mice (Jackson Laboratories, USA) as described [25, 26, 31]. *Ixodes scapularis* larvae was purchased from Tick rearing facility at Oklahoma State University. Larvae were fed on uninfected, or *A. phagocytophilum*-infected mice and the fed larvae were allowed to molt to obtain uninfected or infected tick nymphs. The tick cell line, ISE6, obtained from ATCC, USA, was used throughout this study. Tick cell line was maintained as described in our previous studies [25, 26, 31]. Tick rearing was done in an Environmental Chamber from Parameter Generation and Control, USA. The incubator was set at 23 ± 2 °C with 94% relative humidity and 14:10 light: dark conditions. For noninvasive treatment of ticks, unfed nymphs were immersed in 1x PBS containing the reagent (e.g. XA) for 45 min and washed once with 1x PBS. The wet ticks were applied on a clean paper towel to dry them and only the active ticks were subsequently incubated in the Environmental Chamber for 24 h and further processed for RNA/DNA extractions.

### Cell culture, bacteria, and in vitro infection

Tick cell line ISE6 was maintained in LI5B300 medium as described in our previous studies [25, 26, 31]. Briefly, 2 × 10^5^ cells/well were seeded in cell culture plates one day prior to infection. For in vitro tick cell infections, *A. phagocytophilum* was isolated from HL-60 cultures maintained in IMDM medium. Host cell free *A. phagocytophilum* was isolated as described [25, 26, 31]. Briefly, *A. phagocytophilum*-infected HL-60 cells were centrifuged at 3000 g for 10 min. Cell pellets were resuspended in 1x PBS and incubated in a -80 °C freezer for 10 min to allow increased lysis of cells. Cells were passed through a 27-gauge syringe six-eight times to release the intracellular bacteria. The cell debris was pelleted by centrifugation at 270 g for 3 min and the supernatant was collected. For in vitro cell line infections, 2 × 10^5^ /tick cells/well were infected with 5 MOI *A. phagocytophilum* isolated from infected HL-60 cells in a 12 well cell culture plate. The MOI of isolated *A. phagocytophilum* was calculated based on our previous publication [39]. In the case of XA treatment, 100 µM XA and mock were added 4 h after infection. Tick cells were collected at 24 h post infection and processed for RNA and DNA extractions to evaluate gene expression and bacterial burden, respectively.

### Mice and tick feeding

Ticks were collected from control IgG or EL-6-antibody passively immunized mice from previous study [25] and used in this work. Briefly, 15 µg/mouse of control-IgG, or IsOATP4056-peptide IgG antibodies were injected (intraperitoneal, i.p.) into groups of four C3H/HeN mice one day before tick infestation. After day 1 postimmunization, *A. phagocytophilum*-infected ticks were fed on each group of mice. Completely fed repleted ticks were collected and processed for RNA extractions followed by cDNA synthesis and qRT-PCR analysis. The *ishp* transcripts were analyzed in these ticks and normalized to tick 5.8 S rRNA levels.

### Ethics statement

The protocol for animal work used in this study was approved by the University of Tennessee Institutional Animal Care and Use Committee (IACUC) with permit number 2801 − 0221 and 2801 − 0324. All animal experiments were performed in accordance with the Guide for the Care and Use of Laboratory Animals of the National Institute of Health. Acepromazine was used as a tranquilizer to minimize distress in animals during tick feeding.

### DNA, RNA extractions and quantitative real-time PCR (QRT-PCR)

QRT-PCR was performed as described in our previous publications [25, 26, 31]. *Anaplasma phagocytophilum* loads in tick cells were measured by taking the ratios of absolute amount of bacterial p44 gene copies to that of the respective actin gene copies or 5.8S rRNA gene copies in host cells. Standard curves for QRT-PCR for each gene fragment were prepared starting from 1 ng to 0.00001 ng/ul. DNA was extracted using DNeasy blood and tissue extraction kit (QIAGEN, USA). Transcript loads of tick genes were expressed as the ratios of absolute amounts of individual transcripts to that of tick 5.8S rRNA. Total RNA was extracted using Aurum Total RNA Mini kit (BioRad, USA) and cDNA was prepared using iSCRIPT cDNA synthesis kit (BioRad, USA) or qScript cDNA kit (Quantabio, VWR, USA). QRT-PCR was performed in CFX Opus 96 (BioRad, USA) using Maxima SYBR green qPCR master mix (ThermoScientific, USA). Following are the oligonucleotides used to measure the transcripts of tick *ishp*: FP- 5’ TGACCGAAGAGCACAGACAC 3’ and RP- 5’ CTCCGTCAGTCTTTGGCAGT 3’, tick *ahr*: FP- 5’ CGCACCAAGAGCTACTTCCA 3’ and RP- 5’ TCTCCGTCACAGGTCAGGAT 3’. For detection of *A. phagocytophilum*, we used previously published oligonucleotides [40, 41]. These primers anneal to *A. phagocytophilum p44* genes and can work efficiently to detect this bacterium in ticks [40, 41]. For all other genes, previously published oligonucleotides were used [25–27, 31]. 

### Phylogenetic analysis

IsHP sequence was used as a query sequence in the National Center for Biotechnology Information (NCBI) protein BLAST server. The BLAST analysis revealed hypothetical proteins of several tick species and one mite species. BLAST analysis in human or mouse genomes did not give any significant hits. Hypothetical proteins from 5 tick species and one mite species along with IsHP sequence were selected for multiple sequence alignment and phylogenetic analysis. The sequences were aligned using ClustalW in MegAlign Pro software in DNASTAR and a table showing percentage identity versus percentage similarity was generated. Maximum Likelihood (RAxML) method in DNASTAR was used to construct the tree enabling bootstrap analysis. The settings selected for bootstrap analysis are iterations- 100, seed- default and threads- 2. Bootstrap values more than 70% were considered significant.

### Co-immunoprecipitation assay and LC-MS/MS analysis

Unfed nymphal ticks were lysed in lysis buffer containing 20mM Tris-Cl, pH: 7.5, 110mM NaCl, 1% Triton X-100 and 1mM PMSF followed by centrifuging for 10 min at 16,000 x g at 4 °C and supernatant was collected. For pre-clearing, tick lysate was mixed with 20 µl of protein A/G agarose bead slurry (Pierce, USA) at 4 °C on a shaking incubator for 30 min and the supernatant was collected after centrifuging for 10 min at 16,000 x g at 4 °C. Total tick lysate (150 µg) was mixed with 5 µg of IsOATP4056 antibody (anti-EL-6) or control antibody and incubated at 4 °C for overnight on a shaking incubator Subsequently, 50 µl bead slurry was added and incubated for 4 h at 4 °C on a shaking incubator. The bead slurry was pelleted and washed 3 times with 1X PBS by centrifuging at 16,000 x g for 2 min at 4 °C. 20 µl of 2X Laemmli buffer was added to the bead pellets, boiled for 5 min and supernatants were collected. The supernatants were loaded onto a 12% gel and SDS-PAGE for resolving the protein bands. The gel was stained with Coomassie blue R-250 and a band present only in IsOATP4056 antibody precipitate was excised. The excised protein band was identified by LC-MS/MS analysis performed at MS Bioworks (Ann Arbor, MI, USA).

### Recombinant IsHP protein expression and purification

Full-length IsHP protein purification was performed as described [29, 42]. Full-length *ishp* sequence was amplified using oligonucleotides containing BamHI and XhoI sites. The oligonucleotide sequences are 5’ TG*GGATCC*ATGGCTCCCACGGCGCAGGA 3’ and 5’ GA*CTCGAG*TTAGGGACATCTCTTGGTCT 3’. Full-length PCR product was digested with BamHI-XhoI restriction enzymes and ligated in-frame at BamHI-XhoI digested pGEX-6P-2 vector (Amersham, USA). Empty vector without *ishp* sequence was used as control for purification of recombinant GST protein. Ligation mix was transformed into *E*. *coli* BL21 chemical competent cells and clones were selected on LB agar plates containing ampicillin antibiotic (50 µg/ml). The rHP-GST or rGST proteins were purified from BL21 bacterial cells following instructions from Hook GST protein Spin purification kit-Bacteria (G-Biosciences Inc. USA) as described [29, 42]. Proteins concentration was measured using Pierce BCA protein measurement kit (ThermoFisher Scientific, USA).

### Co-precipitation of IsOATP4056 and Immunoblotting analysis

150 µg of nymphal tick lysate prepared in NP40-based lysis buffer was added to 10 µg of either rIsHP-GST or rGST, made up to 200 µl with 1X PBS and incubated overnight at 4 °C on a shaker. Next day, the mixtures were mixed with 200 µl of glutathione resin and incubated further at 4 °C overnight on a shaker. The resins were pelleted by centrifuging at 2000 x g for 5 min at 4 °C and supernatant was discarded. The resins were washed 3 times with 1X PBS at 4 °C by centrifuging at 2000 x g for 3 min. 20 µl of 4X Laemmli buffer was added to the pelleted resins, boiled for 5 min and supernatants were collected. The supernatants were loaded onto a 12% gel and SDS-PAGE analysis was carried out. Blotting was performed with a modified transfer protocol optimized for low amount of the co-precipitated protein, using Trans-Blot TurboTM (BioRad, USA) by setting the parameters as Voltage-25 V, Current-0.3 A, time-11 min and Towbin’s buffer was used for transfer. Blotted nitrocellulose membranes were blocked with 5% BSA in TBST (0.05% Tween 20) for 1 h at room temperature and incubated overnight in anti-EL6 primary antibody solution at a dilution of 1: 500 (prepared in 5% BSA that is dissolved in TBST). Anti-rabbit secondary antibody conjugated to horse radish peroxidase (Cell Signaling Technologies, USA) was used at a dilution of 1: 5000 and membranes were incubated for 1 h at room temperature. Chemiluminescence was measured using ChemidocTM MP imaging system (BioRad, USA) and manually prepared ECL reagent [1 ml of 250 mM luminol (A8511, Sigma, USA) dissolved in DMSO and 0.44 ml of 90 mM p-Coumaric acid (C9008, Sigma, India) dissolved in DMSO were mixed with 10 ml of 1 M Tris-Cl, pH:8.5. The ECL mixture was stored at -20 °C as 1 ml aliquots. 1 ml of the above aliquot was mixed with 7.5 ml deionized water and stored at -20 °C as 1 ml aliquots of ECL reagent A. 1 ml of ECL reagent A was mixed with ECL reagent B (4 µl of 3% H_2_O_2_) for chemiluminescence detection].

### dsRNA synthesis, transfections, and tick cell line experiments

The mock or dsRNA synthesis was performed as described [24, 29, 31]. Briefly, *ishp*-dsRNA fragment was generated by PCR using 5’ CC*AGATCT*GGCCAAGCCAAGAAAGAGCA 3’ and 5’ CG*GGTACC*GTCTAAATGGTGGCCCAAGG 3’ containing BglII and KpnI sites, respectively. *ahr*-dsRNA fragment was generated by PCR using 5’ CC*AGATCT*AGCAGGGAGCTTCTGTACATC 3’ and 5’ CG*GGTACC*GGATGTCCAACCGCAAGAAC 3’ containing BglII and KpnI sites, respectively. The amplified PCR products were cloned into pL4440 double T7 Script II vector as described [24, 29, 31]. The clones obtained were later processed for dsRNA synthesis using MEGAscript RNAi Kit (Ambion Inc./ThermoFisher Scientific, USA) following manufacturer’s instructions. Transfection of mock and dsRNA into ISE6 tick cells was performed with lipofectamine as described [24, 29, 31]. Briefly, 2 × 10^5^ cells were plated in a 12-well plate in L15B300 medium. After overnight incubation, 750 ng of dsRNA was mixed with 3 μl lipofectamine and was added to each well. After 4 h, 2X L15B300 medium containing 10% percentage of FBS was added and incubated overnight. Following incubation, cell free *A. phagocytophilum* was added and samples were collected after 24 h post infection. Samples were processed for RNA and DNA extraction to quantify the expression of genes and bacterial burden, respectively.

### Preparation of nuclear extracts and electrophoretic mobility shift assay (EMSA)

Nuclear extracts from uninfected or *A. phagocytophilum*-infected ticks were prepared using NE-PER nuclear and cytoplasmic extraction kit (Pierce/Thermo Fisher Scientific, USA) according to manufacturer instructions. Nymphal ticks were bathed in 1X PBS solution containing 1µg *ahR*-dsRNA or 100 µM XA or with respective mock controls for 45 min. Ticks were then dried on paper towels and incubated in environmental chamber for 24 hr. EMSA assays were performed as described [29, 31, 43]. First, we used oligonucleotides 5’ CGGAATTCCGACGCACAACTTTGATGA 3’ and 5’ CCCAAGCTTGCGCTCAGGTCCAGGCA 3’ and amplified a 980 bp PCR fragment containing AhR-binding site from DNA isolated from ticks. Based on the sequencing information, complimentary oligonucleotides consisting of AhR-binding site on *isoatp4056* promoter region 5’ CAACACTTATTTTTCAAAGGTTTGCGTAAGCAAAATCAGTGCTCCGCCTG 3’ and 5’ CAGGCGGAGCACTGATTTTGCTTACGCAAACCTTTGAAAAATAAGTGTTG’ were designed. These oligonucleotides were annealed, and biotin labeled according to Pierce Biotin 3’ End DNA labeling kit (Pierce/Thermo Fisher Scientific, USA). The labeled oligonucleotides were added to a 20 µl reaction mix consisting of reagents from LightShift EMSA optimization and control kit (Pierce/Thermo Fisher Scientific, USA) and nuclear extracts prepared from nymphal ticks. XA was dissolved in 0.5 N NaOH and same solvent without XA was used as a mock-solvent. The mock-solvent or XA-treated or mock-dsRNA or *ahR*-dsRNA-treated unfed *A. phagocytophilum*-infected ticks were generated as described [38]. Nuclear extracts were prepared as described [29, 43]. The entire reactions were incubated at room temperature for 25 min and loaded onto 6% native DNA polyacrylamide gel. The gel was initially pre-run at 100 V with 0.5X Tris-Borate-EDTA and again run with samples at the same settings and conditions. Later, the gel was transferred, UV-cross linked and processed following Chemiluminescent nucleic acid detection module recommendations (Pierce/Thermo Fisher Scientific, USA). Images were captured using ChemiDoc MP imager (BioRad, USA).

### GenBank accession numbers

Following are the GenBank accession numbers used in this study: EEC12410.1 (Conserved hypothetical protein, Ixodes scapularis), KAH6935422.1 (hypothetical protein, *Hyalomma asiaticum*), KAH7955136.1 (hypothetical protein, *Dermacentor silvarum*), KAH7968140.1 (hypothetical protein, *Rhipicephalus sanguineus*), KAK8781104.1 (hypothetical protein, *Amblyomma americanum*), XP_022647163.1 (uncharacterized protein, *Varroa destructor*) and XP_064482138.1 (uncharacterized protein, *Ornithodoros turicata*).

### Statistics

All the data sets were statistically analyzed using GraphPad Prism 9 software (www.graphpad.com). The unpaired t-test with Welch’s correction analysis was carried out to compare the means of experimental data sets. The p values of less than 0.05 were considered as significant.

### Supplementary Information

This article contains supplementary information that includes seven supplementary figures and legends.

## Results

### Identification of IsOATP4056-interacting partner(s) by Immunoprecipitation assay

**Fig. 1:**
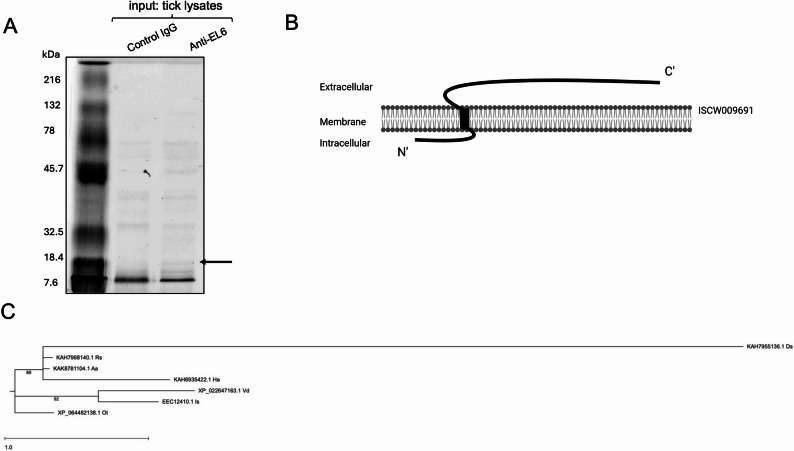
Identification of an IsOATP4056-interacting partner by immunoprecipitation assay. **A**) SDS-PAGE gel image stained with Coomassie blue is shown. Immunoprecipitation was performed with control IgG or IsOATP4056-EL6 antibody (anti EL-6) using uninfected unfed nymphal tick lysates. Arrow indicates band excised for LC-MS/MS analysis. Marker bands are shown in kDa. **B)** A schematic representation (made with BioRender.com) showing organization of ISCW009691 (IsHp) on tick cell plasma membrane based on the prediction by the TMHMM online tool. **C)** Phylogenetic analysis using Maximum Likelihood method and enabling Bootstrap analysis for IsHp was performed using DNASTAR. IsHP amino acid sequence was compared to similar proteins from other ticks and one mite species. Bootstrap values displayed on the phylogenetic tree are above 70% which indicates statistical significance. GenBank accession numbers are indicted. Organism names are indicated as: *Aa- Amblyomma americanum*,* Rs- Rhipicephalus sanguineus*,* Ha-Hyalomma asiaticum*,* Is-Ixodes scapularis*,* Ds-Dermacentor silvarum*,* Vd-Varroa destructor* and *Ot-Ornithodoros turicata*

We performed a co-immunoprecipitation assay with EL-6 antibody and whole tick lysates to pull down any IsOATP4056-interacting partner(s). Co-immunoprecipitation performed with Control-IgG antibody was included as a control in this experiment. The elutes from co-immunoprecipitation experiment were run on SDS-PAGE and stained with Coomassie blue (Fig. 1A). One of the intense protein bands present only in the EL-6 antibody precipitate was excised from the gel and LC-MS/MS was performed to identify the protein. The protein was identified as ISCW009691, a 22 kDa hypothetical protein of *Ixodes scapularis* (Supplementary Fig. 1), designated in this study as IsHP. Prediction of transmembrane helices at TMHMM (https://services.healthtech.dtu.dk/services/TMHMM-2.0/) revealed that IsHP has one short internal loop, one transmembrane domain and one long external loop (Supplementary Fig. 2). The N-terminal end of IsHP is cytosolic, the middle region contain one transmembrane domain and the long C-terminal end is in the extracellular region (Fig. 1B). Multiple sequence alignment analyses revealed that IsHP amino acid sequence shares 30%, 8%, 59%, 57%, 39% and 43% identity with hypothetical or uncharacterized proteins from *Hyalomma asiaticum*,* Dermacentor silvarum*, *Rhipicephalus sanguineus*, *Amblyomma americanum*, *Varroa destructor* and *Ornithodoros turicata*, respectively (Supplementary Fig. 3). Phylogenetic analysis using Maximum Likelihood method and enabling Bootstrap analysis revealed that IsHP amino acid sequence falls within the same clade with *Varroa destructor* mite hypothetical protein (Fig. 1C). The hypothetical proteins from *H. asiaticum*,* D. silvarum*, *R. sanguineus*, *A. americanum* falls within the same clade and hypothetical protein from *O. turicata* forms a different clade (Fig. 1C). Bioinformatic analysis further indicated that IsHP has eight predicted casein kinase II phosphorylation sites, two N-myristoylation sites and two protein kinase C phosphorylation sites (Supplementary Fig. 4).

### IsOATP4056 interacts with Recombinant IsHP

**Fig. 2:**
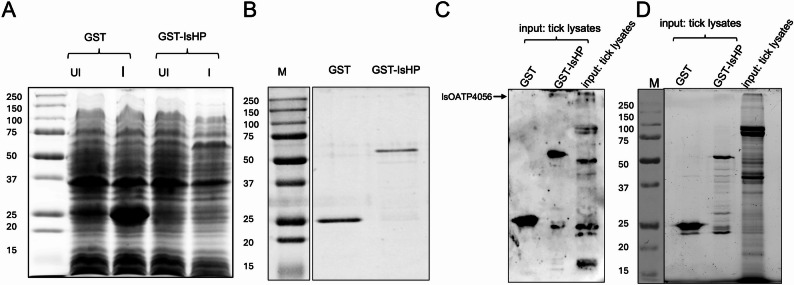
Purification of GST-tagged IsHp and confirmation of its interaction with IsOATP4056. **A)** SDS-PAGE gel image showing expression of rGST and rGST-tagged IsHp after induction with 1mM IPTG (Isopropyl β-D-1-thiogalactopyranoside). UI indicates uninduced and I indicate induced. **B)** SDS-PAGE gel image showing purified recombinant GST and GST-IsHp proteins. **C)** Immunoblot image showing co-precipitation of IsOATP4056 from tick lysates incubated with rGST-IsHp protein. rGST alone was used as a control. IsOATP4056 EL-6 antibody was used to detect IsOATP4056 after immunoblotting. Arrow indicates detection of IsOATP4056 in rGST-IsHp lane and in tick lysate lane used for reference but not in rGST lane. **D**) Total protein profile resulted from TCE staining of the gel run with the samples used in panel C. Marker bands in panel A, B, and C-D are shown in kDa

To further support the immunoprecipitation assay results, we purified recombinant GST-IsHP and GST proteins from *Escherichia coli* bacteria (Fig. 2A and B). SDS-PAGE analysis revealed expected bands for GST-IsHP (~ 50 kDa) and GST (~ 26 kDa) proteins after final protein purification (Fig. 2B). Purified proteins were used to confirm the interaction between IsHP and IsOATP4056. GST or GST-IsHP recombinant proteins were incubated with glutathione resin followed by incubation with unfed nymphal tick total protein lysates or in the reverse order where the resin was added at the end. After washing, the elutes from the resins were run on SDS PAGE. Immunoblotting analysis with EL-6 antibody revealed an expected band for IsOATP4056 above 250 kDa in the whole tick lysates (Fig. 2C and Supplementary Fig. 5A) and in elutes collected from rGST-IsHP (Fig. 2C and Supplementary Fig. 5B). This band was absent in elutes collected from rGST group (Fig. 2C and Supplementary Fig. 5B). TCE-stained gel image shows the elute input from rGST-IsHP and rGST groups (Fig. 2D and Supplementary Fig. 5C). The data from this experiment further confirmed that IsOATP4056 interacts directly with IsHP.

### Expression of *Ishp* is downregulated upon *A. phagocytophilum*-infection but RNAi-mediated Silencing or antibody blocking of IsOATP4056 upregulates its expression in tick cells

**Fig. 3:**
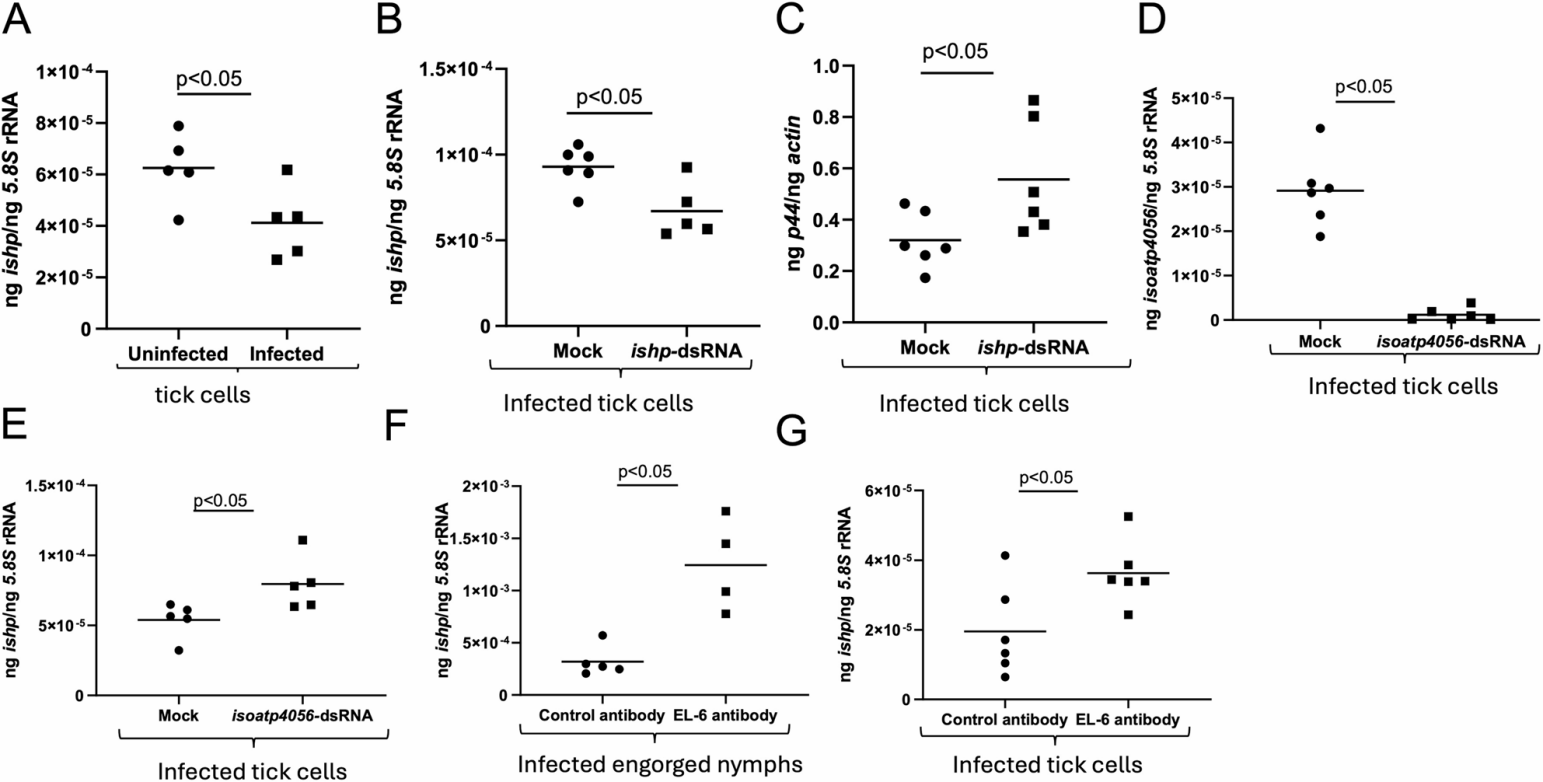
Impairment of *isoatp4056* expression affects *ishp* expression in *A. phagocytophilum*-infected ticks and tick cells. qRT-PCR analysis showing *ishp* transcripts in uninfected or *A. phagocytophilum*-infected tick cells (**A**) and in mock or *ishp*-dsRNA-treated *A. phagocytophilum*-infected (**B**) tick cells. **C**) qPCR showing bacterial loads (*p44* gene levels) in mock or *ishp*-dsRNA-treated *A. phagocytophilum*-infected tick cells. qRT-PCR analysis showing *isoatp4056* (**D**) or *ishp* (**E**) transcripts in mock or *isoatp4056*-dsRNA treated *A. phagocytophilum*-infected tick cells. qRT-PCR analysis showing *ishp* transcripts in *A. phagocytophilum*-infected nymphs fed on control- or EL-6 antibody-immunized mice (**F**) or in *A. phagocytophilum*-infected tick cells treated with control or EL-6 antibody (**G**) at 5 µg/ml. In all panels, each dot represents samples generated from one independent well of a cell culture plate or one individual tick. The *ishp and isoatp4056* transcript levels and *p44* DNA levels were normalized to the 5.8 S rRNA levels. Closed circles indicate uninfected or mock *A. phagocytophilum*-infected groups and closed squares denote infected or *A. phagocytophilum*-infected dsRNA-treated groups. Statistical significance was calculated using unpaired t test with Welch correction. Horizontal bar indicates mean. *P* < 0.05 is considered as significant

Our previous studies showed that *A. phagocytophilum* upregulates IsOATP4056 expression both at RNA and protein levels [25, 31]. Therefore, we first analyzed whether this bacterium has any effect on IsOATP4056 interacting partner, IsHP, expression. Quantitative real-time PCR (qRT-PCR) analysis revealed that *A. phagocytophilum* infection downregulates *ishp* transcripts in tick cells (Fig. 3A). We then silenced *ishp* expression to evaluate this effect on bacterial burden in tick cells. qRT-PCR analysis showed significantly (*P* < 0.05) low levels of *ishp* transcripts in *ishp*-dsRNA-treated *A. phagocytophilum*-infected tick cells when compared to the levels noted in mock-dsRNA-treated control cells (Fig. 3B). We noted significantly (*P* < 0.05) increased bacterial burden in *ishp*-dsRNA-treated tick cells compared to the burden noted in mock-dsRNA-treated tick cells (Fig. 3C). Furthermore, we tested whether silencing of *isoatp4056* expression has any effect on *ishp* transcript levels. qRT-PCR analysis revealed significantly lower levels of *isoatp4056* transcript levels in *isoatp4056*-dsRNA-treated *A. phagocytophilum*-infected tick cells compared to the levels noted in mock-treated controls (Fig. 3D). We also noted significantly (*P* < 0.05) higher levels of *ishp* transcripts in *isoatp4056*-dsRNA-treated *A. phagocytophilum*-infected tick cells compared to the levels noted in mock-treated control (Fig. 3E). Furthermore, *ishp* transcript levels were noted to be significantly (*P* < 0.05) increased in *A. phagocytophilum*-infected nymphs that were fed on IsOATP4056-peptide passively immunized (EL-6 antibody-injected) mice compared to the levels noted in ticks fed on control IgG immunized mice (Fig. 3F). In addition, we also noted significantly (*P* < 0.05) increased *ishp* transcripts upon treatment of *A. phagocytophilum*-infected tick cells with IsOATP4056 EL-6 antibody compared to the levels noted in control IgG treated control cells (Fig. 3G). These results indicate that IsOATP4056 could negatively impact *ishp* expression in ticks.

### RNAi-mediated Silencing of *Ishp* expression downregulates a toll pathway component in tick cells

**Fig. 4:**
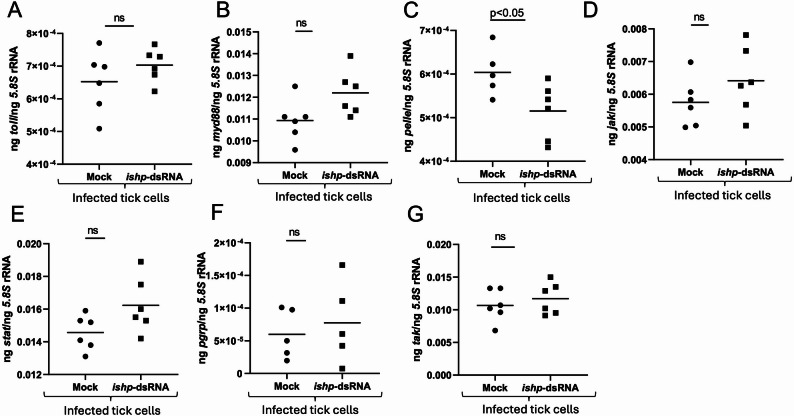
RNAi-mediated silencing of *ishp* expression affects the expression of some of the innate immune genes in *A. phagocytophilum*-infected tick cells. qRT-PCR analysis showing expression of *toll* (**A**), *myd88* (**B**), *pelle* (**C**), *jak* (**D**), *stat* (**E**), *pgrp* (**F**) and *tak* (**G**) in mock or *ishp*-dsRNA-treated *A. phagocytophilum*-infected tick cells. In all panels, each dot represents samples generated from one independent well of a cell culture plate. The transcript levels *of* immune genes were normalized to the 5.8 S rRNA levels. Closed circles indicate mock groups and closed squares denote *A. phagocytophilum*-infected dsRNA-treated groups. Statistical significance was calculated using unpaired t test with Welch correction. Horizontal bar indicates mean. *P* < 0.05 is considered as significant

Our previous study reported that blocking of IsOATP4056 with a EL-6 antibody in *A. phagocytophilum*-infected tick cells resulted in increased expression of immune genes *myd88* and *pelle* [25]. We therefore tested whether blocking of *ishp* expression with RNAi-mediated silencing has any effect on tick innate immune gene expression (Fig. 4). qRT-PCR analysis showed no significant (*P* >0.05) differences in the expression of *toll* (Fig. 4A), *myd88* (Fig. 4B), *jak* (Fig. 4D), *stat* (Fig. 4E), *pgrp* (Fig. 4F) and *tak* (Fig. 4G) transcripts between mock- or *ishp*-dsRNA-treated-*A. phagocytophilum* infected tick cells. However, we noted that *pelle* transcripts were significantly (*P* < 0.05) downregulated in *ishp*-dsRNA-treated-*A. phagocytophilum* infected tick cells compared to the levels noted in mock-treated controls (Fig. 4C). These results show that RNAi-mediated knockdown of *ishp* expression specifically downregulates *pelle* expression in *A. phagocytophilum*-infected tick cells.

### RNAi-mediated Silencing of *isoatp4056* expression upregulates the transcripts of some of the innate immune genes in tick cells

**Fig. 5:**
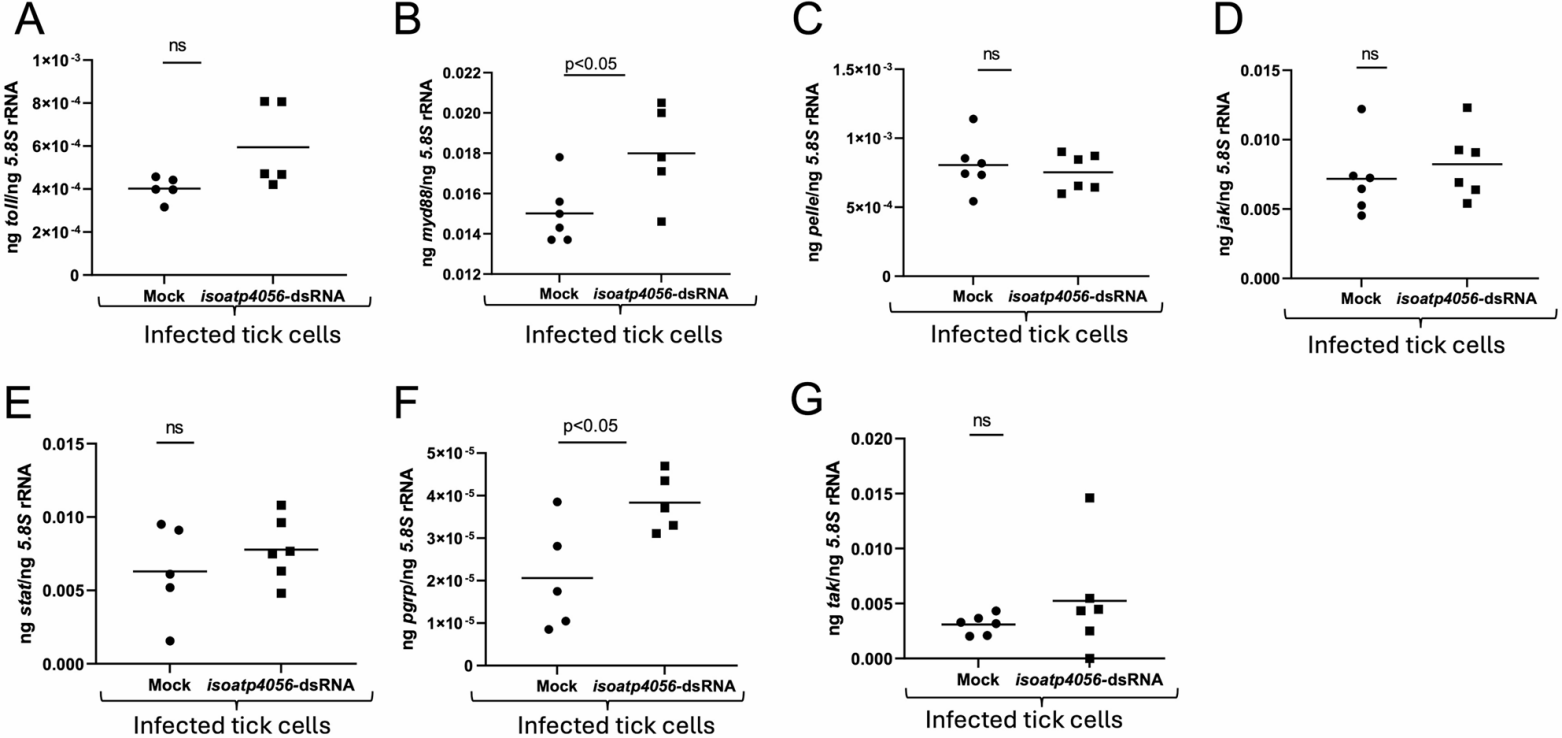
RNAi-mediated silencing of *isoatp4056* expression affects the expression of some of the innate immune genes in *A. phagocytophilum*-infected tick cells. qRT-PCR analysis showing expression of *toll* (**A**), *myd88* (**B**), *pelle* (**C**), *jak* (**D**), *stat* (**E**), *pgrp* (**F**) and *tak* (**G**) in mock or *isoatp4056*-dsRNA-treated *A. phagocytophilum*-infected tick cells. In all panels, each dot represents samples generated from one independent well of a cell culture plate. The transcript levels *of* immune genes were normalized to the 5.8 S rRNA levels. Closed circles indicate mock groups and closed squares denote *A. phagocytophilum*-infected dsRNA-treated groups. Statistical significance was calculated using unpaired t test with Welch correction. Horizontal bar indicates mean. *P* < 0.05 is considered as significant

We then tested whether blocking of *isoatp4056* expression with RNAi-mediated silencing has any effect on tick innate immune gene expression. qRT-PCR analysis showed no significant (*P* > 0.05) differences in the expression of *toll* (Fig. 5A), *pelle* (Fig. 5C), *jak* (Fig. 5D), *stat* (Fig. 5E), and *tak* (Fig. 5G) transcripts between mock- or *isoatp4056*-dsRNA-treated-*A. phagocytophilum* infected tick cells. However, we noted that *myd88* (Fig. 5B) and *pgrp* (Fig. 5F) transcripts were significantly (*P* < 0.05) upregulated in *isoatp4056*-dsRNA-treated-*A. phagocytophilum* infected tick cells when compared to the levels noted in mock-treated controls. These results show that in contrast to silencing of the *ishp* expression, RNAi-mediated knockdown of *isoatp4056* expression upregulated the transcripts of some of the innate immune genes in *A. phagocytophilum*-infected tick cells.

### Tryptophan metabolite xanthurenic acid (XA) affects Aryl hydrocarbon receptor (AhR) expression in tick cells

**Fig. 6:**
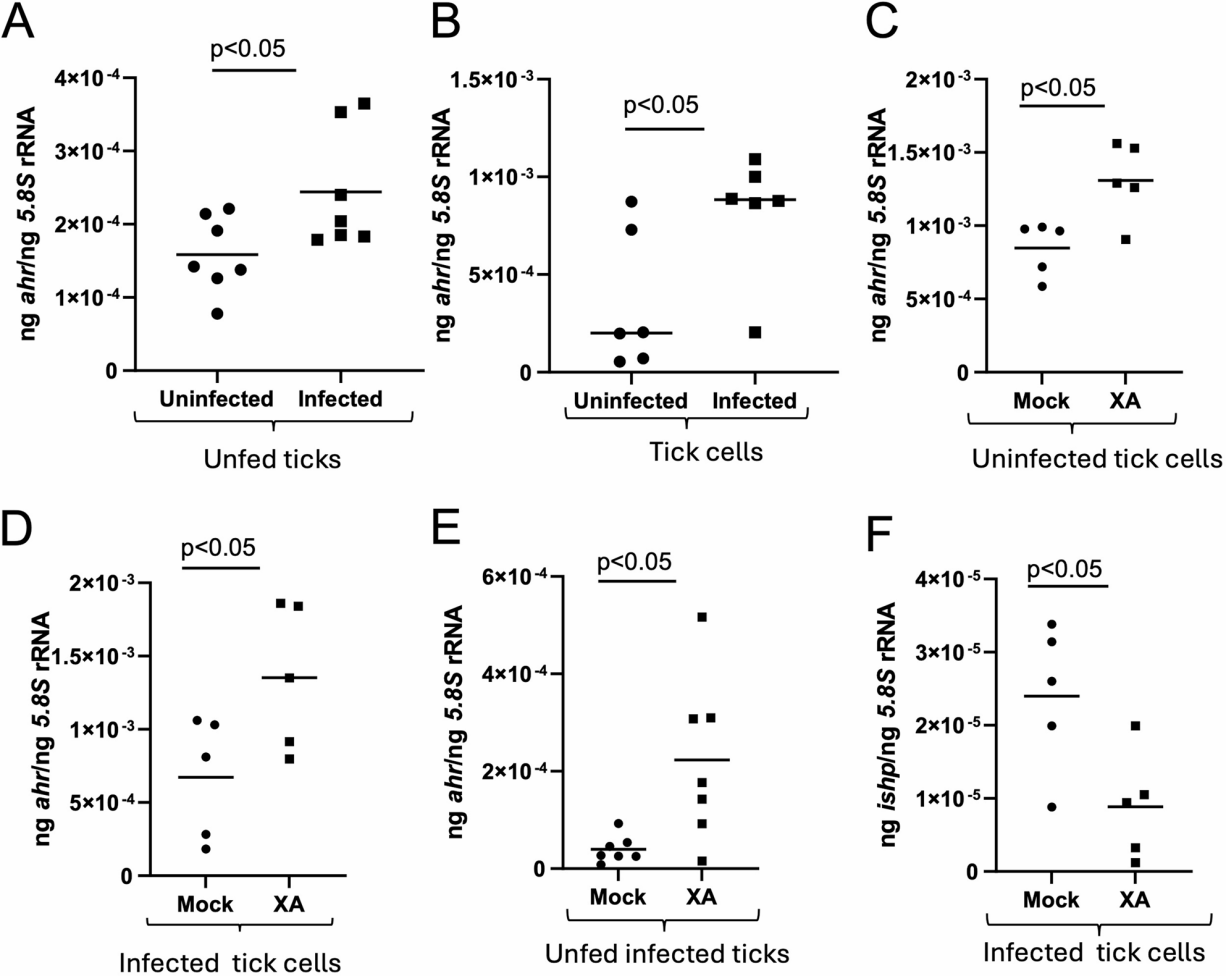
Expression of Aryl hydrocarbon receptor (AhR) is upregulated upon *A. phagocytophilum* infection and XA treatment in ticks and tick cells. qRT-PCR analysis showing *ahr* transcripts in uninfected or *A. phagocytophilum*-infected ticks (**A**) or tick cells (**B**). qRT-PCR analysis showing expression of *ahr* transcripts in mock- or XA-treated uninfected tick cells (**C**) or *A. phagocytophilum*-infected tick cells (**D**) or in unfed *A. phagocytophilum*-infected ticks (**E**). (**F**) qRT-PCR analysis showing *ishp* transcripts in mock- or XA-treated *A. phagocytophilum*-infected tick cells. In all panels, each dot represents samples generated from one independent well of a cell culture plate or one individual tick. The transcript levels of *ahr* or *ishp* were normalized to the 5.8 S transcript levels. Closed circles indicate uninfected, or *A. phagocytophilum*-infected mock groups and closed squares denote infected or *A. phagocytophilum*-infected XA-treated groups. Statistical significance was calculated using unpaired t test with Welch correction. Horizontal bar indicates mean. *P* < 0.05 is considered as significant

In our previous study, we noted that *isoatp4056* promoter activation is influenced by XA [31]. AhR is a cytosolic receptor and transcription factor that translocate to the nucleus after binding to its ligands [44]. Tryptophan pathway metabolites such as kynurenic acid and XA are known to be ligands that activate AhR [44, 45]. qRT-PCR analysis revealed that arthropod *ahr* transcripts were significantly (*P* < 0.05) upregulated in *A. phagocytophilum*-infected unfed nymphs when compared to the levels noted in unfed uninfected nymphs (Fig. 6A). Similar observation was noted in *A. phagocytophilum*-infected tick cells at 24 post infection (p.i.) (Fig. 6B). qRT-PCR analysis also revealed that *ahr* transcripts were significantly upregulated in uninfected (Fig. 6C) or *A. phagocytophilum*-infected (Fig. 6D) tick cells and in unfed *A. phagocytophilum*-infected ticks (Fig. 6E) upon XA treatment when compared to transcript levels noted in respective mock-treated controls (Figs. 6C-E). Furthermore, we noted significantly (*P* < 0.05) reduced *ishp* transcript levels upon treatment of *A. phagocytophilum*-infected tick cells with XA compared to the levels noted in mock-treated control. These results show that XA influences *ahr* expression in ticks and tick cells.

### RNAi-mediated Silencing of *Ahr* expression downregulates *isoatp4056* but upregulates *Ishp* expression affecting *A. phagocytophilum* burden in tick cells

**Fig. 7:**
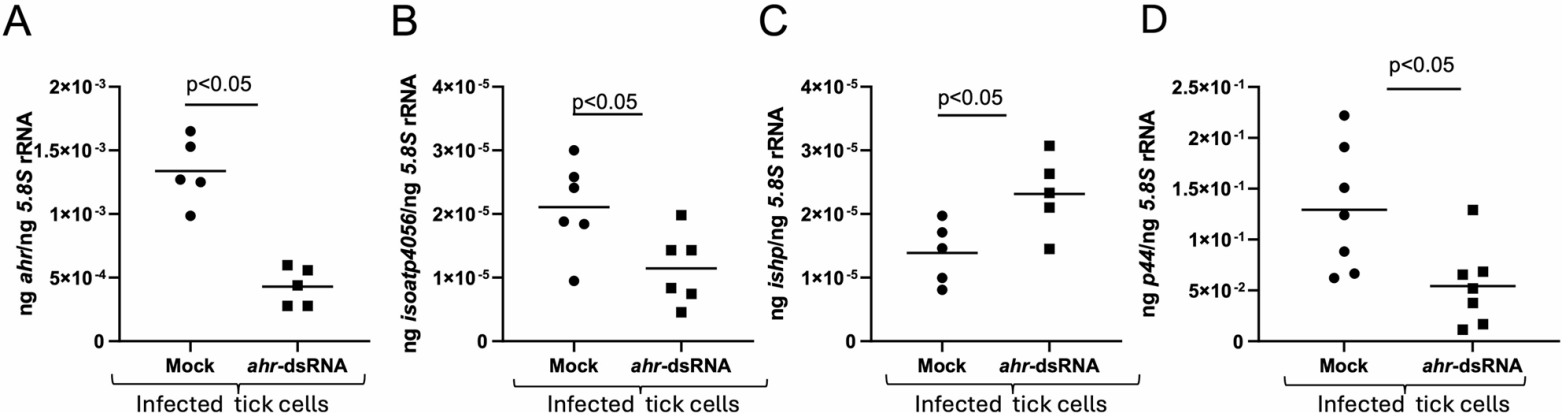
RNAi-mediated silencing of *ahr* expression affects *isoapt4056* expression and bacterial burden in *A. phagocytophilum*-infected tick cells. qRT-PCR analysis showing expression of *ahr* (**A**), *isoatp4056* (**B**) *and ishp* (**C**) and bacterial loads represented by p44 gene levels (**D**) in mock or *ahr*-dsRNA-treated *A. phagocytophilum*-infected tick cells. In all panels, each dot represents samples generated from one independent well of a cell culture plate. The transcript levels of *ahr*,* isoatp4056*,* ishp* or *p44* DNA levels were normalized to the 5.8 S rRNA levels. Closed circles indicate mock groups and closed squares denote *A. phagocytophilum*-infected *ahr-dsRNA*-treated groups. Statistical significance was calculated using unpaired t test with Welch correction. Horizontal bar indicates mean. *P* < 0.05 is considered as significant

We then tested whether RNAi-mediated silencing of *ahr* expression has any effect on *isoatp4056* and *ishp* transcripts. qRT-PCR analysis showed significantly (*P* < 0.05) reduced levels of *ahr* transcripts in *ahr*-dsRNA-treated *A. phagocytophilum*-infected tick cells compared to the levels noted in mock-treated controls (Fig. 7A). In addition, we noted significantly (*P* < 0.05) reduced *isoatp4056* transcript levels (Fig. 7B) and significantly (*P* < 0.05) increased *ishp* transcript levels (Fig. 7C) in *ahr*-dsRNA-treated *A. phagocytophilum*-infected tick cells compared to the levels noted in mock-treated controls. We also noted that upon *ahr* expression silencing (Supplementary Fig. 6), *A. phagocytophilum* burden was significantly (*P* < 0.05) reduced in *ahr*-dsRNA-treated *A. phagocytophilum*-infected tick cells compared to the levels noted in mock-treated controls (Fig. 7D). These results show that *ahr* silencing affects expression of *isoatp4056* and *ishp* transcripts. Taken together, results from figures six and seven indicate that XA-mediated increase of *ahr* expression will upregulate *isoatp4056* expression resulting in the inhibition of *ishp* expression.

### *Anaplasma phagocytophilum* infection results in activation of *isoatp4056* promoter via XA and AhR binding

**Fig. 8:**
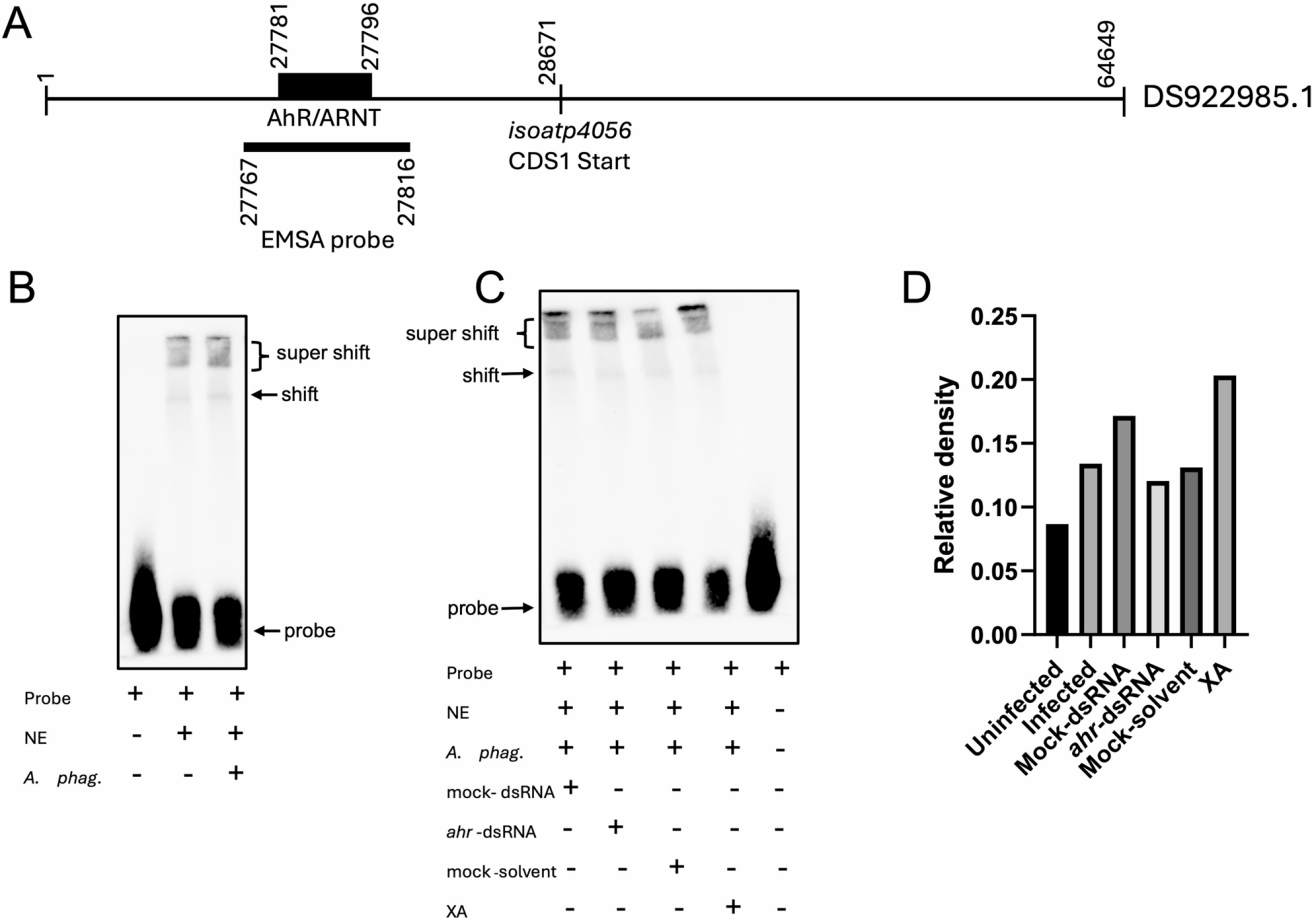
AhR binds *isoatp4056* promoter region. **A)** A schematic representation showing *isoatp4056* promoter region in *I*. *scapularis* whole genome shotgun sequence (GenBank accession number DS922985.1). The rectangular box above the bold line indicates the predicted AhR/ARNT binding site. Location and position of the sequence used to generate EMSA probe is indicated as a grey bar below the bold line. The start position of *isoatp4056* CDS1 is shown on the bold line. The schematic representation of genomic region is not to the scale. **B)** Gel shift assays with biotinylated *isoatp4056* promoter probe containing AhR/ARNT binding site and nuclear proteins (1.5 µg) from uninfected or *A*. *phagocytophilum*–infected nymphs are shown. **C)** Gel shift assays with biotinylated *isoatp4056* promoter probe containing AhR/ARNT binding site and nuclear proteins (1.5 µg) from *A*. *phagocytophilum*–infected nymphs bathed in mock-dsRNA or *ahr-*dsRNA and mock-solvent or 100 µM XA are shown. NE indicates nuclear extracts and + or - indicates presence or absence, respectively. **D**) Quantification of gel shift band intensities normalized to the respective probe intensities in B and C is shown

To determine whether AhR binds to *isoatp4056*-promoter, we performed electrophoretic mobility shift assays (EMSA) with a probe designed based on the predicted AhR-binding site in the genome region (GenBank acc. no. DS922985.1) of *isoatp4056* gene (Fig. 8). We noted the presence of a putative AhR-binding site from position 27,781–27,796 (Fig. 8A). We first sequenced this region and noted a nucleotide change in the putative AhR-binding site (Supplementary Fig. 7A). Therefore, based on the sequencing information a synthetic probe was designed covering 50 bp around this region. The EMSA assay results showed an increased shift when *ahr* probe was incubated with nuclear extracts generated from *A. phagocytophilum*-infected unfed ticks compared to shift noted when probe was incubated with nuclear extracts generated from uninfected unfed ticks (Fig. 8B and D). Using RNAi method, we further silenced *ahr* expression in unfed *A. phagocytophilum*-infected ticks (Supplementary Fig. 7B). We noted decreased shift when *ahr* probe was incubated with nuclear extracts generated from *A. phagocytophilum*-infected-*ahr*-dsRNA-treated unfed ticks compared to shift noted when probe was incubated with nuclear extracts generated from mock-treated control ticks (Fig. 8C and D). In addition, we noted increased shift when *ahr* probe was incubated with nuclear extracts generated from *A. phagocytophilum*-infected-XA- treated unfed ticks compared to the shift noted when probe was incubated with nuclear extracts generated from mock-solvent-treated control ticks (Fig. 8C and D). These results indicate that AhR and XA could activate *isoatp4056* promoter. Collectively, these results indicate that AhR and XA binding activates *isoatp4056* expression that negatively impacts *ishp* expression and innate immune gene expression leading to *A. phagocytophilum* survival in tick cells.

## Discussion

Protein-protein interactions during host-pathogen interplay facilitates the survival of pathogens in host cells [46–48]. Our previous studies provided evidence on the interplay between arthropod IsOATP4056 and tryptophan pathway to control innate immune responses for the survival of *A. phagocytophilum* in ticks [24–27, 31]. In this study, we provide evidence that IsOATP4056 directly interacts with IsHP, a tick transmembrane protein, to elicit this effect.

Immunoprecipitation analysis revealed multiple bands when tick extracts were incubated with anti-EL6 antibody. We selected only one band that had high intensity in the pull-down reaction with anti-EL6 antibody. This band was absent in the pull-down reaction performed with control IgG antibody. LC-MS/MS analysis revealed this protein as IsHP. The immunoprecipitation data was supported with direct co-precipitation with recombinant IsHP protein. These two different assays confirmed a direct interaction between IsOATP4056 and IsHP. Vertebrate OATPs and OATs are known to interact with PDZ [Postsynaptic density protein 95 (PSD-95), *Drosophila* disc large tumor suppressor (Dlg), and Zonula occludens-1 protein (ZO-1)] domain-containing proteins [49, 50]. Therefore, it is not surprising to hypothesize that tick OATPs could also interact with several other proteins in the vector host. The appearance of multiple bands in pull-down experiment supports this view. Future studies will unravel the significance of the interactions of tick IsOATP4056 with other proteins.

IsOATP4056 is a transmembrane protein with six extracellular loops (EL) that are exposed outside of the plasma membrane [25]. EL-6 is the last extracellular loop region on the IsOATP4056 protein [25]. TMHMM prediction also revealed that IsHP is also a transmembrane protein with one transmembrane domain and most of the C-terminal residues present in the extracellular loop that are outside the cell surface (Fig. 1B). We believe that the extracellular loop regions of IsOATP4056 could interact with extracellular loop region of IsHP. The presence of long regions of extracellular loops in both proteins could facilitate these interactions. Our data suggests that IsOATP4056 could directly interact with IsHP and functionally block the activation of innate immune signaling by the latter molecule.

The primary amino acid (aa) sequence of IsHP consists of 204 aa and in GenBank this protein is annotated as a conserved hypothetical protein (Acc. no. EEC12410.1). BLAST analysis of IsHP revealed that N-terminal region of 148 aa is identical with *I. scapularis* cilia- and flagella-associated protein 251-like (435 aa, Acc. no. XP_042145890.1) and sarcalumenin (707 aa, Acc. no. XP_002408077). The human cilia- and flagella-associated protein 251 (CFAP251) is important in the motility of cells and in the formation of mitochondrial sheath around axonemes during flagellogenesis [51]. In addition, human sarcalumenin is a calcium binding protein located in sarcoplasmic reticulum and regulates the calcium uptake [52]. The high identity of IsHP amino acid sequence at the N-terminal region with these proteins indicates that this protein may also have a function in cell motility and/or in calcium signaling. Studies have elucidated that calcium is an important second messenger that influences innate immune signaling [53, 54]. The role of IsHP in modulating calcium signaling to activate innate immune responses in ticks cannot be ruled out.

A recent report has shown that AhR modulates immune responses in mosquitoes [55]. In addition, a study has reported that knockout of AhR in mice resulted in the downregulation of various vertebrate OATPs including *oatp1a1*, *oatp1b2*, and *oatp2b1* [44]. The observation of reduced transcripts of *isoatp4056* upon *ahr* silencing supports a conserved role for AhR in regulating OATPs in mammalian and arthropod host. Furthermore, our EMSA results indicate that arthropod AhR could directly bind to the *isoatp4056* promoter region to regulate this gene expression. Our EMSA results also suggests that XA could further enhance AhR binding to the *isoatp4056* promoter region.

**Fig. 9:**
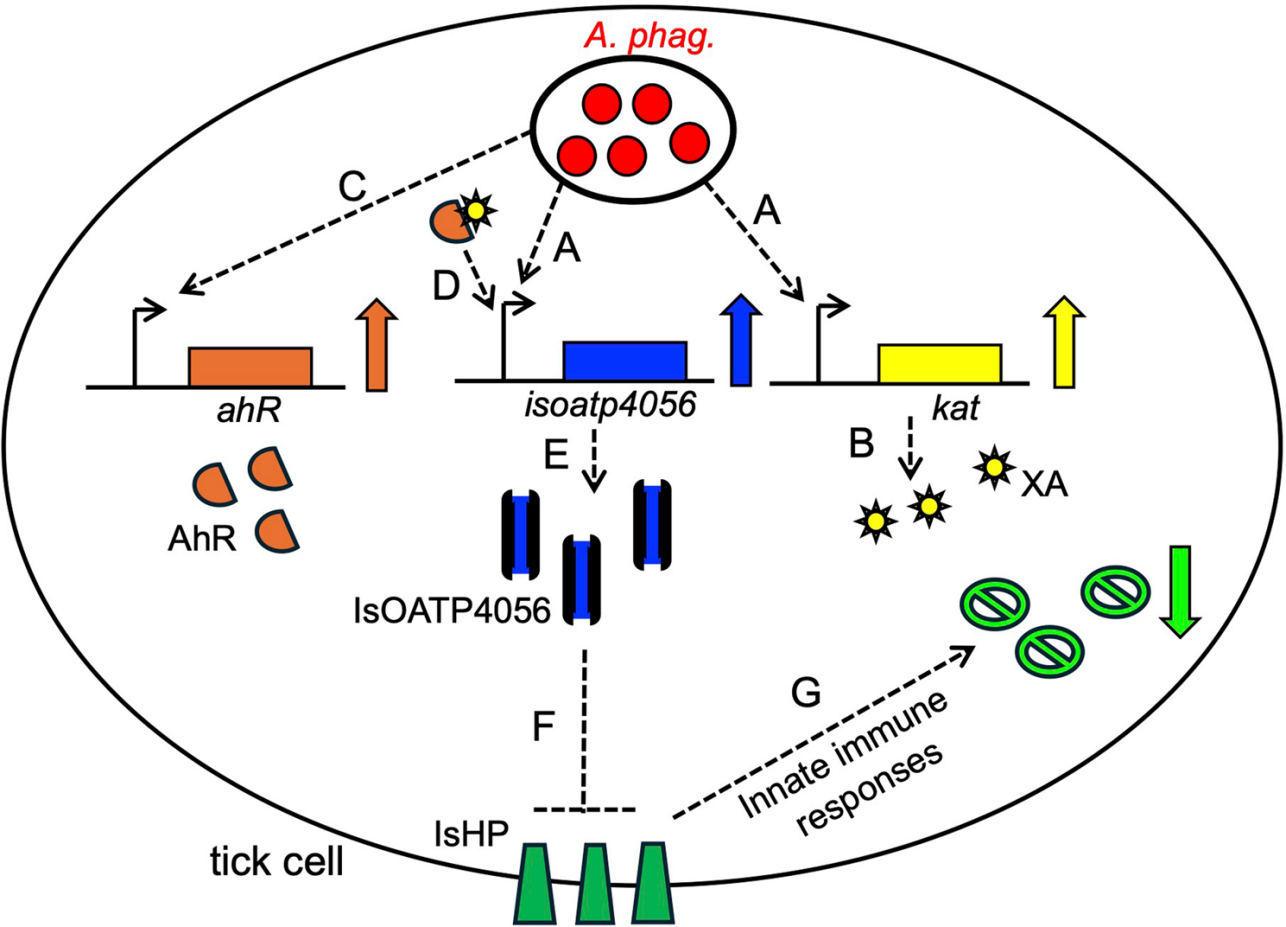
A schematic model showing interplay between IsOATP4056 and IsHp in tick cells. **A**) *Anaplasma phagocytophilum* infection upregulates *isoatp4056* and *kat* transcripts. **B)** Upregulation of *kat* transcripts leads to increased production of XA. **C)** Bacterial infection also leads to increased production of AhR. **D**) Increased AhR and XA levels could lead to their pronounced binding to regulate *isoatp4056* gene expression. **E)** Increased *isoatp4056* transcripts leads to increased IsOATP4056 protein levels. **F)** IsOATP4056 binds and inhibits IsHP function. **G)** Inhibition of IsHP impairs innate immune responses to limit bacterial infection. These events lead to successful bacterial survival in tick cells

The observations on the effect of RNAi-mediated knockdown of *isoato40456* expression on increased *ishp* and innate immune gene expression that affected *A. phagocytophilum* growth in tick cells indicate that IsOATP4056 is upstream of IsHP and negatively impacts expression of the latter molecule. In addition, the direct binding of IsOATP4056 with IsHP could affect the function of the latter molecule. The observation on the effect of silencing of *ishp* expression on innate immune gene expression supports the view that IsHP is involved in the regulation of this pathway. We noted that RNAi-mediated silencing of *ishp* expression was modest compared to the silencing of *isoatp4056* expression. This observation suggests that IsHP might be a likely essential protein for the activation of innate immune responses in ticks to control *A. phagocytophilum* infection. Based on the current and previous findings, we propose a model demonstrating interplay among molecules that we studied (Fig. 9). Upon *A. phagocytophilum* infection, *isoatp4056* and *kat* transcripts are increased (Fig. 9A) [31]. The increase in *kat* transcripts will lead to increased production of XA (Fig. 9B) [26]. *Anaplasma phagocytophilum* also upregulates transcriptional molecule AhR (Fig. 9C). Availability of increased XA will lead to activation of AhR binding to *isoatp4056* promoter (Fig. 9D). The increased binding of AhR will lead to production of more IsOAT4056 (Fig. 9E). IsOATP4056 then binds to IsHP and negatively regulates its function (Fig. 9F). IsHP is a positive regulator of innate immune gene expression. Binding of IsOAT4056 to IsHP will inhibit the role of the latter molecule and affects innate immune gene expression (Fig. 9G). The suppression of arthropod innate immune gene expression will lead to survival of *A. phagocytophilum* in tick cells.

In summary, our study provides a detailed analysis of arthropod molecular pathways related to the transporter protein IsOATP4056 that are modulated by an obligate intracellular pathogen *A. phagocytophilum* for its survival in the vector host.

## Supplementary Information

Supplementary Material 1.

## Data Availability

Data is provided within the manuscript or supplementary information files.
