# Supplementary material for: An arthropod transporter IsOATP4056 exerts inhibitory effect on its interacting membrane protein to facilitate rickettsial pathogen survival in ticks
Source: Cell Commun Signal. 2025 Nov 22;23:540. doi: 10.1186/s12964-025-02545-w (PMC12750571; doi:10.1186/s12964-025-02545-w)
Supplement: Supplementary file 1 — Supplementary Material 1. [file 12964_2025_2545_MOESM1_ESM.pdf]

An arthropod transporter IsOATP4056 exerts inhibitory effect on its interacting membrane protein to facilitate rickettsial pathogen survival in ticks

P P Mahesh [1], Hameeda Sultana [1], and Girish Neelakanta [1, *]

Supplementary information

Supplementary figure legends

Supplementary Figure 1. LC-MS/MS results for the gel band from co-immunoprecipitation assay. LC-MS/MS data for the band excised from SDS-PAGE gel (for image shown in Figure 1A) is shown. The results indicated several peptides matches to IsHp. All matched peptides are highlighted with orange color.

Supplementary Figure 2. TMHMM prediction for IsHp. The TMHMM prediction of IsHP amino acid sequence indicated that 1-12 residues are inside (shown in blue color) the membrane, 13-31 residues are in transmembrane region (shown in red color) and 32-204 residues are exposed outside the membrane (shown in pink color).

Supplementary Figure 3. Percentage identity for IsHp and similar proteins in different tick species and one mite species. The amino acid sequence of IsHP was aligned with amino acid sequences of proteins from other ticks and mites. The alignment was performed using

CLUSTALW in DNASTAR. The percent identity boxes were generated based on this alignment. Values above black boxed diagonal line indicates percent identity and values below black boxed diagonal line indicates percent similarity. GenBank accession numbers are also shown.

Supplementary Figure 4. Number of post-translational modifications on IsHP. The IsHP primary amino acid sequences was submitted for PROSITE scan for posttranslational modifications. The modified site, modified residue, and the type of post-translational modifications are shown.

Supplementary Figure 5. IsOATP4056 interacts with IsHP. A) Immunoblot image showing detection of IsOATP4056 in tick lysates is shown for reference. Arrow indicates IsOATP4056 band. B) Immunoblot image showing co-precipitation of IsOATP4056 from tick lysates incubated with rGST-IsHp protein. rGST alone was used as a control. IsOATP4056 EL-6 antibody was used to detect IsOATP4056 after immunoblotting. C) Total protein profile resulted from TCE staining of the gel run with the samples used in panel B. In each panel, marker bands are shown in kDa. This data is from an independent experiment other than the data shown in Figure 2.

Supplementary Figure 6. RNAi-mediated silencing efficiency of *ahr* expression. qRT-PCR analysis showing expression of *ahr* in mock or *ahr*-dsRNA-treated *A. phagocytophilum*-infected tick cells. Each dot represents samples generated from one independent well of a cell culture plate. The transcript levels of *ahr* were normalized to the 5.8S transcript levels. Closed circles

indicate *A. phagocytophilum*-infected mock group and closed squares denotes *ahr-dsRNA*-treated group. Statistical significance was calculated using unpaired t test with Welch correction. Horizontal bar indicates mean. P<0.05 is considered as significant.

Supplementary Figure 7. RNAi-mediated silencing efficiency of *ahr* expression in unfed A. *phagocytophilum*-infected ticks. A) Genomic sequence of a reference GenBank deposited sequence and the sequence from this study for AhR probe design is shown. Colored region indicates AhR-binding site. **B)** qRT-PCR analysis showing expression of *ahr* in mock or *ahr*-dsRNA-treated unfed *A. phagocytophilum*-infected ticks. Each dot represents samples generated from one tick. The transcript levels of *ahr* were normalized to the 5.8S transcript levels. Closed circles indicate *A. phagocytophilum*-infected unfed nymphal mock group and closed squares denotes *ahr-dsRNA*-treated group. Statistical significance was calculated using unpaired t test with Welch correction. Horizontal bar indicates mean. P<0.05 is considered as significant.

LC-MS/MS peptide mapping

tr|B7Q0N7|B7Q0N7_IXOSC (100%), 22,274.4 Da
Putative uncharacterized protein OS=Ixodes scapularis GN=IscW_ISCW009691 PE=4 SV=1
4 exclusive unique peptides, 4 exclusive unique spectra, 6 total spectra, 40/204 amino acids (20% coverage)

M A P T A Q E K V S   V D A R G V L I M I   V A L A G I A A L W   V N E F M P A D D G
F D P S T Y V Y R S   P Q S P H K G H S R   E A T R E P P K P V   L L S L E D C G K **L**
A Q D A V R E Y V Q   R H A A E P R D D S   A E D S A P T D D D   E A S K A R **D Y E E**
A S M T E E H R H G   G S K A A A R H A E   A E D T S A Q S E E   E H E E A H A P E L
A D S E E V K A K P   R **K S K P V A Q T E**   **K** E L P K **T D G D A**   **P K P A K K** A K K T
K R C P

Supplementary Fig. 1

Analysis of IsHP amino acid sequence at TMHMM

TMHMM result

```
# WEBSEQUENCE Length: 204
# WEBSEQUENCE Number of predicted TMHs:  1
# WEBSEQUENCE Exp number of AAs in TMHs: 19.72675
# WEBSEQUENCE Exp number, first 60 AAs:  19.72675
# WEBSEQUENCE Total prob of N-in:        0.77204
# WEBSEQUENCE POSSIBLE N-term signal sequence
WEBSEQUENCE     TMHMM2.0        inside       1    12
WEBSEQUENCE     TMHMM2.0        TMhelix     13    31
WEBSEQUENCE     TMHMM2.0        outside     32   204
```

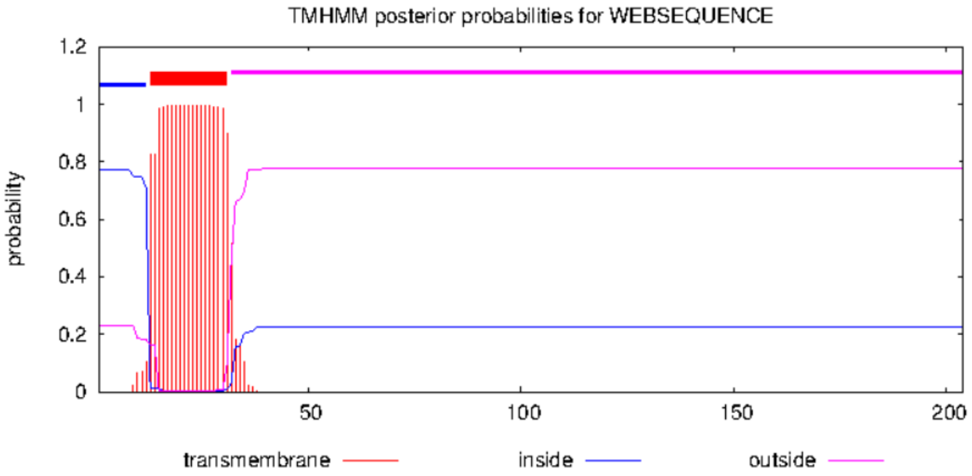

Supplementary Fig. 2

		A	B	C	D	E	F	G
A	EEC12410.1 Is		30.12	8.43	59.04	56.63	38.55	43.37
B	KAH6935422.1 Ha	42.17		8.43	46.99	46.99	16.87	26.51
C	KAH7955136.1 Ds	14.46	15.66		7.23	7.23	9.64	8.43
D	KAH7968140.1 Rs	74.7	55.42	13.25		89.16	36.14	49.4
E	KAK8781104.1 Aa	69.88	54.22	14.46	93.98		36.14	45.78
F	XP_022647163.1 Vd	61.45	38.55	19.28	60.24	59.04		33.73
G	XP_064482138.1 Ot	60.24	37.35	15.66	62.65	62.65	57.83	

Supplementary Fig. 3

IsHP modifications-PROSITE scan

Residues	Modified residue	Modification
4-7	4	Casein kinase II phosphorylation
73-76	73	Casein kinase II phosphorylation
100-103	100	Casein kinase II phosphorylation
107-110	107	Casein kinase II phosphorylation
122-125	122	Casein kinase II phosphorylation
148-151	148	Casein kinase II phosphorylation
179-182	179	Casein kinase II phosphorylation
186-189	186	Casein kinase II phosphorylation
130-135	-	N-Myristoylation
131-136	-	N-Myristoylation
179-181	179	Protein kinase C phosphorylation
200-202	200	Protein kinase C phosphorylation

Supplementary Fig. 4

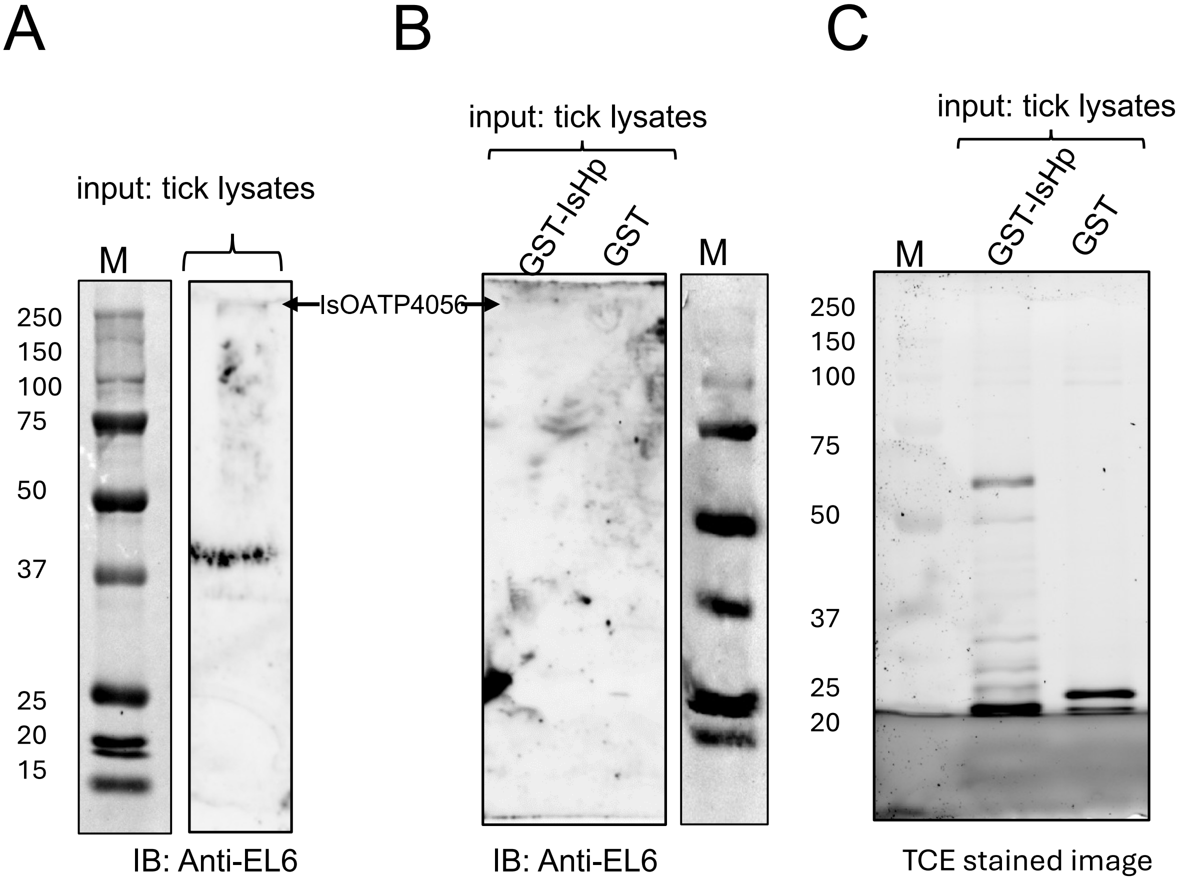

Supplementary Fig. 5

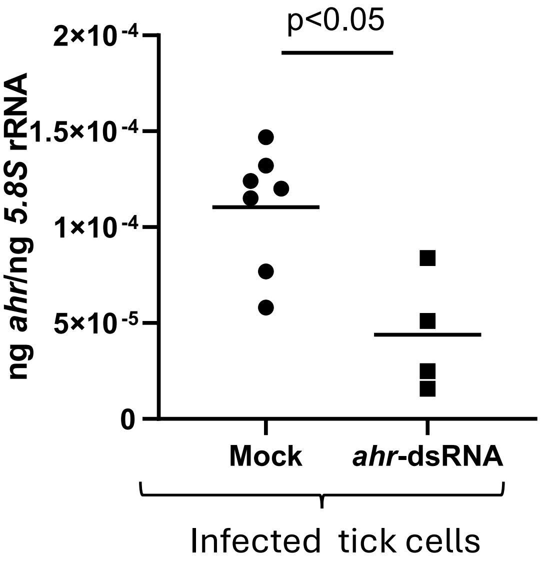

Supplementary Fig. 6

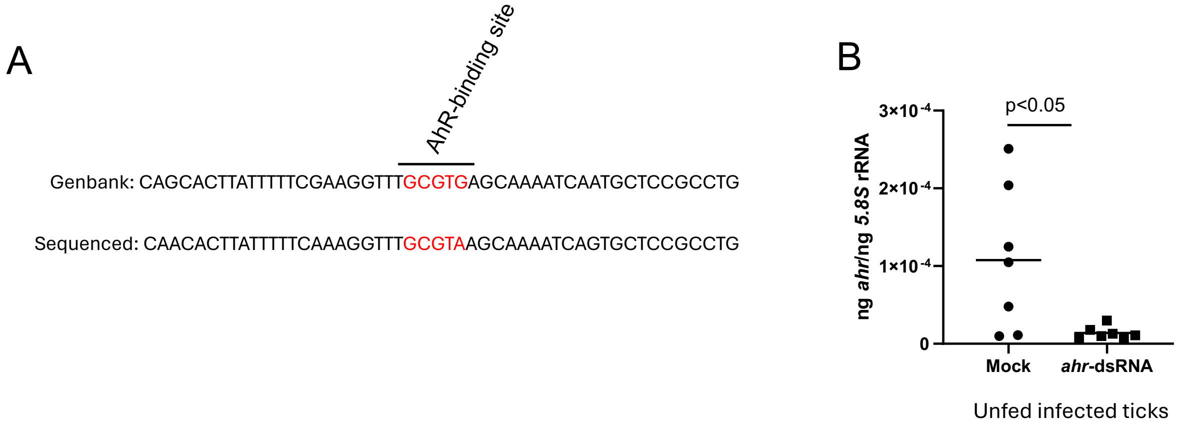

Supplementary Fig. 7
